# Gut Microbiota and Short-Chain Fatty Acids in the Pathogenesis of Necrotizing Enterocolitis in Very Preterm Infants

**DOI:** 10.32607/actanaturae.27623

**Published:** 2025

**Authors:** E. N.Kukaev, A. O. Tokareva, O. A. Krogh-Jensen, A. A. Lenyushkina, N. L. Starodubtseva

**Affiliations:** National Medical Research Center for Obstetrics Gynecology and Perinatology Named after Academician V.I. Kulakov of the Ministry of Healthcare of Russian Federation, Moscow, 117997 Russia; V.L. Talrose Institute for Energy Problems of Chemical Physics, N.N. Semenov Federal Research Center for Chemical Physics, Russian Academy of Sciences, Moscow, 119991 Russia; Federal State Autonomous Educational Institution of Higher Education I.M. Sechenov First Moscow State Medical University of the Ministry of Health of the Russian Federation (Sechenov University), Moscow, 119048 Russia; Moscow Institute of Physics and Technology, Dolgoprudny, Moscow region, 141701 Russia

**Keywords:** necrotizing enterocolitis, very premature infants, diagnostics, gas chromatography-mass spectrometry, short-chain fatty acids, gut microbiota

## Abstract

The development of a symbiotic gut ecosystem is a crucial step in postnatal adaptation. The gut microbiome of very preterm infants is characterized by an overall instability, reduced microbial diversity, and a predominance of Gram-negative Proteobacteria, all factors associated with an increased risk of necrotizing enterocolitis (NEC). Short-chain fatty acids (SCFAs) are the key bacterial metabolites that are essential for maintaining intestinal homeostasis, supporting immune development, enhancing intestinal barrier integrity, and reducing inflammation. This review examines the role of gut microbiota and SCFAs in neonatal NEC, with a focus on potential diagnostic and therapeutic strategies. Clinical studies have consistently demonstrated a significant decrease in total SCFA levels and individual bacterial metabolites in preterm infants with NEC. This finding has been corroborated by various experimental models. Clarification of the role of SCFAs in NEC pathogenesis, determination of their diagnostic utility, and assessment of the feasibility of developing comprehensive pro- and postbiotic formulations require multi-center, multi-omics investigations that include a large cohort of very preterm infants.

## INTRODUCTION

Necrotizing enterocolitis (NEC) is a severe gastrointestinal disease that affects newborns. It is characterized by marked inflammation of the intestinal wall, followed by necrosis and potential perforation. NEC is a significant gastrointestinal complication that primarily affects premature infants, with rare instances reported in infants born after 32 weeks gestation [1]. The primary factors that predispose to excessive gut inflammation include gastrointestinal immaturity, impaired bacterial colonization, and the lack of enteral feeding with breast milk [2]. The incidence of NEC is inversely proportional to gestational age, ranging from 2% to 10% in very preterm infants (28–32 weeks of gestation) and reaching 55% among extremely preterm newborns [3].

Notwithstanding the progress achieved in modern medicine, the incidence of NEC has not changed substantially over time in very low birth weight (VLBW) infants and constitutes a major cause of adverse outcomes in this cohort. The need for surgical intervention due to intestinal perforation or suspicion thereof in NEC ranges from 20% to 52%. Mortality rates for VLBW infants with surgical NEC in developed countries average 30%, reaching 50–72% among extremely low birth weight (ELBW) newborns [4, 5]. According to clinical observations, the progression of NEC is especially unfavorable in cases with concurrent congenital pneumonia, hemodynamically significant patent ductus arteriosus, and ELBW [6]. Between 22.7% and 35% of newborns who survive the surgical stage of NEC will develop intestinal failure syndrome, a condition with a reduction in gut function below the level necessary for the absorption of macronutrients and/or water and electrolytes [7]. Not only does the occurrence of NEC have implications for the gastrointestinal tract, but it also substantially increases the chances of adverse neurological outcomes. Due to the large surface area, considerable vascularization, and high concentration of immune cells, inflammation in the intestine can lead both to intestinal wall perforation and development of systemic effects that impact other tissues and organs [8]. Prospective cohort studies examining the long-term outcomes of NEC reveal that 37.6–56.8% of preterm infants weighing less than 1,000 g at birth experience delayed neuropsychiatric development, a percentage significantly greater than in those without NEC [9].

Given the high prevalence of NEC and the significant risk of adverse outcomes, the development of innovative predictive and diagnostic tools is a priority in the study of this disease. Identifying early specific biomarkers of NEC opens the possibility of detecting excessive inflammatory processes in the gut even before the onset of clinical symptoms [10]. Such an approach would allow for the timely identification of high-risk groups, which, in turn, would ensure early initiation of conservative treatment and the prospective implementation of targeted innovative therapies. Particularly important is the use of noninvasive predictive and diagnostic methods that eliminate phlebotomy losses and painful stimuli, which is critical for VLBW newborns. One such method is the determination of fecal calprotectin levels as a potential early biomarker of NEC in preterm infants. However, its diagnostic value remains a subject of scientific debate [11].

Over the past twenty years, the gut microbiome has been the focus of considerable research, owing to its significance in maintaining health and its connection to diverse diseases like diabetes mellitus, asthma, and inflammatory bowel diseases, including NEC [12]. The reduced frequency of inflammatory diseases in individuals with an abundance of SCFA-producing bacteria and higher SCFA concentrations has stimulated active research in this field [13]. The analysis of gut microbiota composition involves costly and timeconsuming tools, such as 16S rRNA sequencing, which generates extensive and complex datasets that are difficult for clinicians to interpret. Therefore, quantitative analysis of microbial metabolic activity via fecal SCFA levels using gas chromatography-mass spectrometry (GC-MS) merits particular consideration [14, 15]. GC-MS affords rapid, accurate, and non-invasive analysis, making it ideal for neonatal intensive care units (NICUs), notably for very premature infants at elevated risk of NEC.

This review aims to summarize and analyze current data on the pathogenetic role of SCFAs, key metabolites of the gut microbiota, in the development of NEC in very preterm infants (less than 32 weeks of gestation). This avenue appears promising both for understanding the pathogenesis of the disease and for developing novel diagnostic and preventive strategies.

## GUT MICROBIOTA IN THE PATHOGENESIS OF NEC

The bacterial community of the gastrointestinal tract is a vast population of microorganisms (approximately 10^12^–10^14^ cells), representing between 100 and 1,000 various species. Because of the ability to influence various bodily functions by releasing thousands of substances into the bloodstream, this ecosystem is frequently referred to as the “second genome” or even the “second brain” [14].

The microbiome of a newborn develops from birth, affected by factors such as the method of delivery (vaginal delivery or cesarean section), feeding regimen (breast or formula feeding), and the environment [16]. Initially highly plastic and variable, the composition of the neonatal microbiota stabilizes in early childhood [17]. Prolonged hospitalization, antibiotic therapy, feeding via oro- or nasogastric tubes, lack of contact with maternal microflora, and a deficiency of breast milk are key factors influencing the specific development of gut microbiota in preterm infants, the microbial profile of which differs from that of fullterm newborns [18]. Gastrointestinal tract colonization in infants within neonatal intensive care units (NICU) is thought to result in a reduced alpha-diversity of the microbial community with simultaneous enrichment in genes responsible for antibiotic resistance [19, 20, 21, 22].

In very preterm infants, the gut is mostly populated by opportunistic facultative anaerobes, such as Proteobacteria and Firmicutes representatives. This coincides with a reduction in the prevalence of commensal bacteria, including Actinobacteria and Bacteroidota [23, 24]. A significantly increased relative abundance is observed of bacteria of the Enterobacteriaceae family, which comprises Enterobacter, Escherichia, and Klebsiella (phylum Proteobacteria). In contrast, beneficial bacteria of the genus Bifidobacterium (phylum Actinobacteria) are markedly underrepresented compared to full-term infants [23, 24]. Of particular interest is the observation that during the development of the microbial community in very preterm infants a transition from the dominance of one genus of bacteria to another occurs, reflecting the high dynamism and instability of the microbiota in such infants [22, 25, 26]. These changes could be associated with external influences, including antibiotic administration and feeding specifics [22]. Due to the significant instability in the gut microbiome of preterm infants, coupled with the small study group sizes, it is challenging to pinpoint the specific bacteria linked to the onset of NEC [26].

Prior research indicated decreased gut microbiome diversity in very preterm infants, with an even more substantial reduction observed in infants diagnosed with NEC [27]. It should be noted that the reduction in commensal bacteria, specifically representatives of the genera *Bifidobacterium* (phylum Actinobacteria) and *Bacteroides* (phylum Bacteroidota), and the increase in opportunistic microorganisms of the phylum Proteobacteria (especially the family Enterobacteriaceae) and Firmicutes (genera Staphylococcus, Clostridium, Streptococcus, and Blautia) become increasingly pronounced [27, 28, 29, 30, 31]. A correlation between Gammaproteobacteria, particularly the Enterobacteriaceae family, and NEC development was demonstrated in a major longitudinal study of preterm infants with a birth weight under 1,500 g [31]. An increase in the abundance of potentially pathogenic Gammaproteobacteria with a simultaneous decrease in Bacteroides abundance prior to NEC manifestation has also been confirmed in other works [27, 32, 33].

Stewart C.J. et al. (2016) suggested that the etiology of NEC in very preterm infants should be interpreted in terms of the instability of the developing intestinal microbiome rather than specific microorganisms as potential pathogens [25]. This instability is manifested in frequent transitions between dominant bacterial communities. A longitudinal investigation into the gut microbiome of 35 very preterm infants demonstrated that NEC was only observed when bacteria from the genera Klebsiella and Escherichia (family Enterobacteriaceae, phylum Proteobacteria), or Staphylococcus and Enterococcus (phylum Firmicutes), predominate. Moreover, NEC did not occur in the presence of a more diverse bacterial community with a high relative abundance of Bifidobacterium. These data suggest that, in the pathogenesis of NEC, one should consider not only (and not primarily) the factor of colonization by a specific pathogenic bacterial species, but more broadly, the factor of microbiome stability and diversity, which underscores the multifactorial nature of this pathology [25].

**Fig. 1:**
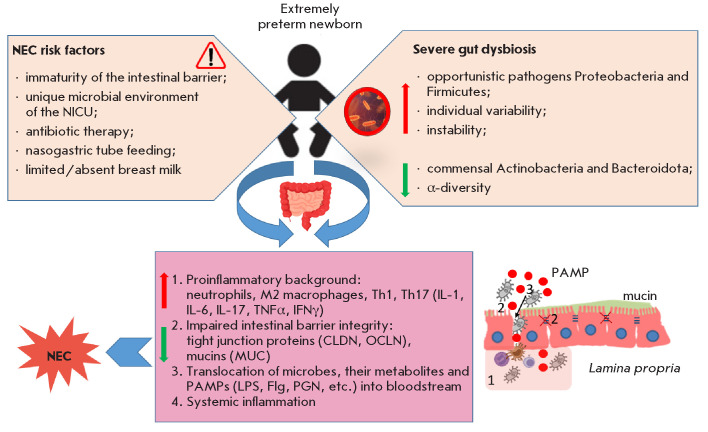
Scheme of NEC pathogenesis in very preterm infants

The immature gut of very preterm infants exhibits impaired epithelial cell differentiation, fewer Paneth cells, and a reduction in the synthesis of protective mucus [34]. Insufficient formation of intestinal mucus, immature gut immunity, and reduced endogenous production of antimicrobial factors can lead to increased bacterial adhesion and heightened exposure to bacterial endotoxin (lipopolysaccharide, LPS) from Gram-negative bacteria (specifically the phylum Proteobacteria prevalent in such children). This stimulates Toll-like receptors 4 (TLR4) on epithelial cells, leading to their apoptosis and the disruption of intestinal epithelial barrier integrity, as well as triggering a pronounced inflammatory reaction mediated by TNFα, IL-1β, and other pro-inflammatory cytokines [35]. Ultimately, these processes increase the risk of developing neonatal sepsis or a local inflammation (Fig. 1) [6, 36, 37].

In NEC, a pathogen may not always be identified [25]. Diagnosis is established through clinical and radiological indicators, negating the need for microorganism isolation. However, examining the contribution of microbiome dysregulation to the etiology of NEC is essential in integrating the microbiome analysis into clinical application. Early detection of bacterial overgrowth, particularly of species associated with NEC and late-onset sepsis, as well as analysis of changes in the structure of the microbiome over time could be a promising avenue in treating very preterm infants [36]. Particular emphasis is placed on the microbiological analysis of mucosal secretions from the upper respiratory and gastrointestinal tracts within the first 24 h of life in identifying early neonatal sepsis and NEC risk factors in very preterm infants [38]. Yet incorporating data on the gut microbiota and its changes into daily clinical practice is hindered by several obstacles, encompassing the substantial diversity and intricacy of microbiota composition, as well as the absence of standardized analytical methodologies and procedures. These factors complicate the interpretation of the data obtained and require further research.

Conventional methods used to study microbiota involve culturing microorganisms in various nutrient media. These methods facilitate a thorough examination of live bacterial cultures and functional assessments, like antibiotic sensitivity. However, such methods have significant limitations: only a small percentage of gut microorganisms can be cultured in a laboratory setting, and the culturing process can be time-consuming. Consequently, culture-based methods reflect the species composition of the microbiota to a limited extent and provide qualitative, rather than quantitative, data [39].

Polymerase chain reaction (PCR), a potent and extremely sensitive molecular technique, is employed to amplify and identify specific DNA segments to rapidly detect particular microorganisms. However, PCR is primarily employed to detect known species and does not facilitate an assessment of species diversity and richness within microbiota composition [40].

The use of next-generation sequencing (NGS) of 16S rRNA has become widespread in microbiota studies, because it allows for the analysis of the genetic material of both culturable and non-culturable microorganisms [40]. This method allows one to identify bacterial species, estimate their relative abundance, and evaluate their potential metabolic activity. However, 16S rRNA NGS has its drawbacks, such as the difficulty in differentiating closely related species, a lack of detailed information on the functional aspects of bacterial communities, high cost and time requirements for the analysis, and the complexity of data processing and interpretation (Table 1) [41].

**Table 1:** Comparison of major methods for investigating microbiota composition: advantages and limitations

Method	Advantages	Limitations
Culture-based methods	Obtaining live cultures; Conducting functional assessments, including antibiotic sensitivity tests.	Limited number of culturable species; Laborintensive and time-consuming process; Primarily qualitative data [39].
PCR	High sensitivity; Rapid detection of established microorganisms.	The detection is limited to established microorganisms; The total species diversity cannot be estimated [40].
16S rRNA sequencing	Analysis of culturable and non-culturable species; Assessment of relative abundance; Microbiota profiling.	The lack of discrimination between closely related species; Limited functional data; High cost; Interpretation challenges [40, 41].

Despite providing comprehensive data on microbial community composition and genetic profiles, 16S rRNA sequencing does not fully elucidate the functional role of each species within the microbiome. Microorganisms are capable of adapting by altering enzyme synthesis levels and activity, allowing them to influence the environment, community members, cells, and the host organism itself. Multi-omics approaches integrating metagenomics, metatranscriptomics, proteomics, and metabolomics are being developed to address these challenges. These methods can provide more comprehensive data concerning the intensity of gut dysbiosis, interaction dynamics, and the metabolic functions of the microbial community, which could be especially beneficial in clinical research and in managing complex diseases linked to changes in the microbiome [42].

## SHORT-CHAIN FATTY ACIDS ARE KEY METABOLITES OF THE GUT MICROBIOTA

Bacteria are capable of synthesizing approximately 15,000 molecules unique to the human host, for which immune and intestinal cells express specific receptors [43]. Bacterial metabolites can penetrate the intestinal barrier and exert systemic effects [44, 45]. Notable among these bioactive compounds of microbial origin are antimicrobial peptides, conjugated linoleic acid, gamma-aminobutyric acid (GABA), and short-chain fatty acids (SCFAs) [45].

In the human body, these compounds perform a multitude of important functions, including inhibiting the synthesis of pro-inflammatory cytokines, maintaining the integrity of the intestinal epithelial barrier, and stimulating the proliferation and differentiation of colonocytes [45]. SCFAs serve as universal energy substrates for various cells, with butyric acid acting as the preferred energy source for colonocytes, providing 60–70% of their energy requirements [46]. Compared to individual microorganisms or their combinations, SCFAs, the end products of bacterial metabolism, offer a more insightful indication of gut status. Understanding the mechanisms of action of these metabolites is fundamental in identifying potential NEC biomarkers and developing new therapeutic targets.

Of the SCFAs produced in the gut, only 5% are detected in feces, as colonocytes absorb the majority [47]. The average concentration of SCFAs in feces ranges from units to tens of mmol/kg [48]. Only a small fraction (approximately 1%) of SCFA is absorbed into the portal vein as salts [49]. Acetic acid (acetate) is a key end product of glycolysis in a multitude of commensal microorganisms, including representatives of Lactobacillus, Clostridium, Blautia (phylum Firmicutes), Bacteroides and Prevotella (phylum Bacteroidota), as well as Bifidobacterium (phylum Actinobacteria) [30]. Propionic acid (propionate) is synthesized by a limited number of gut bacteria via the metabolism of succinate, acrylate, and propanediol. These microorganisms include representatives of the genera Clostridium, Veillonella (phylum Firmicutes), Propionibacterium (phylum Ac tinobac teria ), and Bacteroides (phylum Bac teroido ta ) [4]. Bacteria producing butyric acid (butyrate) mainly belong to the phylum Firmicutes, including the families Ruminococcaceae, Lachnospiraceae, Erysipelotrichaceae, and Clostridiaceae [50]. Butyrate production is notably high in species belonging to the class Clostridia [51]. Additionally, the gut microbiota includes bacteria that employ the metabolic byproducts of other microorganisms to produce butyric acid, thereby inhibiting the buildup of lactate and acetate [5]. For example, acetate produced by Bifidobacterium is converted into butyrate by bacteria of the class Clostridia [52].

Acetic acid functions as a fatty acid biosynthesis substrate, is involved in the Krebs cycle [53], and demonstrates anti-inflammatory activity [54]. Propionate contributes to the improvement of barrier function and intestinal epithelial integrity and also plays an important role in regulating glucose and lipid homeostasis in the liver [55]. Butyric acid (butyrate) is a key energy source for the epithelial and immune cells of the large intestine [56, 57], increases the expression of tight junction proteins, thereby promoting the maintenance of gut barrier integrity [56], and exhibits a pronounced anti-inflammatory effect [58].

Establishing the relationship between gut microbiota and health requires reliable quantitative tools for determining metabolite concentrations in various biological matrices, such as plasma, serum, urine, and feces. Contemporary research uses such techniques as capillary electrophoresis (CE), nuclear magnetic resonance (NMR), and liquid and gas chromatography coupled with mass spectrometry (LC-MS and GC-MS) [59, 60, 61, 62, 63, 64]. Mass spectrometry is the method of choice for the quantitative analysis of low-molecular-weight compounds owing to its high sensitivity and specificity [59, 60, 63, 64, 65, 66]. However, the application of these methods to the analysis of SCFAs in feces is not without some challenges. Firstly, the high lipid content in feces reduces the extraction efficiency of water-soluble compounds. Secondly, the volatile and partially hydrophilic properties of SCFAs significantly complicate their analysis via LC-MS, which requires multistep sample preparation, including extraction and derivatization stages [67]. Besides making the analysis more complex and time-consuming, it also elevates the technical variation [68]. GC-MS is a reliable method for the quantitative determination of low-molecular-weight compounds [64, 69, 70]. Due to the volatile nature of SCFAs, their analysis via GC-MS can be performed without derivatization if one uses chromatographic columns with highly polar phases and specific liquid-liquid extraction conditions [59, 69].

The precision and reliability of quantitative assessments could be compromised when examining infant fecal samples relative to adult samples. This is attributable to the significant fluctuations in water content, challenges in standardizing sample weight, the application of diapers and defecation stimulants within the NICU, and the scarcity of available biological material.

**Table 2:** Comparative characteristics of methods for the quantitative determination of SCFAs in feces

Analysis method	Principle	Advantages	Limitations
Capillary electrophoresis [61]	Separation of ions in an electric field within a capillary	– Method simplicity – Low reagent consumption – Possibility of analyzing multiple compounds simultaneously	– Low sensitivity – Requires a high degree of sample purification
NMR spectroscopy [62]	Registers magnetic properties of atomic nuclei	– Non-destructive method – Simultaneous analysis of many metabolites – No need for derivatization	– Low sensitivity – High equipment cost
LC–MS [60, 63, 65, 66]	Separation of compounds in the liquid phase followed by ion analysis	– High sensitivity and specificity – Wide range of detectable SCFAs – Possibility of isotopic normalization	– Multi-step sample preparation – Need for derivatization and/or use of internal standards – Volatile compound issues
GC–MS [59, 62, 64, 69, 71]	Separation of volatile compounds in gas phase and their mass spectrometric analysis	– High accuracy and sensitivity – Suitable for the analysis of volatile SCFAs – Possibility of analysis without derivatization (under optimal conditions)	– Time-consuming sample preparation – Need for derivatization for non-volatile compounds – Variable reproducibility when analyzing neonatal feces

Table 2 presents a comparative analysis of the most commonly employed analytical methods for determining SCFAs in fecal matter. The predominant methodologies employed in clinical and research environments are presented, with particular attention to their relevance in the context of neonatal fecal sample analysis. Special attention is paid to parameters such as sensitivity, specificity, sample preparation requirements, and the potential limitations of each method. Considering the instability of SCFAs, GC-MS, when appropriately configured, may be the superior option, notwithstanding sample preparation needs.

## MECHANISMS OF ACTION OF SCFAs

The primary function of SCFAs involves providing energy to intestinal cells, including colonocytes and cells of the immune system, thereby stimulating their metabolism, proliferation, and differentiation [72, 73]. Butyric acid (butyrate), one of the key SCFAs, reverses mitochondrial respiration deficits and prevents autophagy in energy-deprived germ-free colonocytes [74]

SCFAs play a crucial role in maintaining intestinal barrier integrity, preventing the development of increased intestinal permeability syndrome, known as “leaky gut.” This syndrome facilitates bacterial translocation, intensifies inflammatory processes, and may cause systemic complications [75]. Adding butyrate to neuroglioma epithelial cells (H4) and including it in the diet of mice increases local oxygen consumption, which stabilizes the hypoxia-inducible factor (HIF). As a result, the transcription of the genes involved in the synthesis of key tight junction components, such as mucin (MUC20), claudins (CLDN2, 4, 11, and 15), and occludin (OCLN), is activated [76]. These alterations contribute to the fortification of intercellular connections, thus impeding the passage of pathogens through the intestinal barrier.

SCFAs play a key role in the regulation of the immune response in the gut, making them important mediators of the interaction between the microbiota and the immune system [45]. Butyrate induces colonocytes to synthesize the anti-inflammatory cytokine IL-18, which not only enhances mucin production but also stimulates the synthesis of antimicrobial peptides, including defensins and cathelicidins, which are effective against numerous pathogens [77]. Concurrently, the bacterial metabolite inhibits IL-1- induced expression of pro-inflammatory genes, such as IL-6, CX3CL1, and CXCL5, thus regulating microbiota composition and preventing dysbiosis [78, 79].

SCFAs, acting as ligands for G-protein-coupled receptors (GPR43/FFAR2, GPR41/FFAR3), play a role in the activation and differentiation of cells in both innate and adaptive immunity [58, 80]. Butyrate guides macrophage differentiation toward the M2 immunosuppressive phenotype, which contributes to the suppression of inflammation and the preservation of tissue homeostasis [81]. Acetic acid reduces intestinal inflammation by activating the GPR43/FFAR2 receptor on granulocytes, contributing to a reduction in their inflammatory activity [82].

SCFAs influence gene expression levels in immune cells via epigenetic mechanisms. For example, butyrate and propionate inhibit histone deacetylases (HDACs), which promotes chromatin decondensation and the activation of transcription for the genes responsible for immunoregulation [72, 83]. Through HDAC inhibition, butyrate directs T-cell differentiation into Foxp3+ regulatory T cells (Tregs) [84]. Tregs play a central role in suppressing excessive immune responses to commensal microorganisms and preventing the development of chronic inflammation. In addition, the activation of GPR43 and GPR109A receptors on dendritic cells by butyrate enhances Treg differentiation through the upregulation of the anti-inflammatory cytokine IL-10 [85].

SCFAs are also known to affect how B-cell functions. Kim M. et al. (2016) demonstrated that SCFAs, particularly acetate, directly stimulate bacterial differentiation of B cells into IgA-producing cells [86]. The secretion of immunoglobulin A (IgA) provides protection to the intestinal mucosa, preventing the overgrowth of pathogenic microorganisms and protecting commensals [86].

SCFAs inhibit the growth and colonization of pathogenic intestinal microflora, including the microorganisms of the family Enterobacteriaceae, such as E. coli, K. pneumoniae, and P. aeruginosa [87]. They alter intracellular pH levels in pathogens, creating unfavorable conditions for their survival. This is attributable to the capacity of SCFAs to permeate the bacterial cell in a non-ionized state, subsequently dissociating, thereby decreasing intracellular pH and compromising metabolic functions within pathogen cells [88].

In colonocytes, butyrate activates the peroxisome proliferator-activated receptor-gamma (PPARγ), which plays an important role in regulating fatty acid metabolism. The activation of PPARγ stimulates β-oxidation, resulting in heightened oxygen consumption by colonocytes, consequently diminishing oxygen availability within the intestinal lumen. The change in the oxygen environment hinders the growth of aerobic pathogenic microorganisms, especially those of the Enterobacteriaceae family, which need oxygen for proliferation [89].

**Fig. 2:**
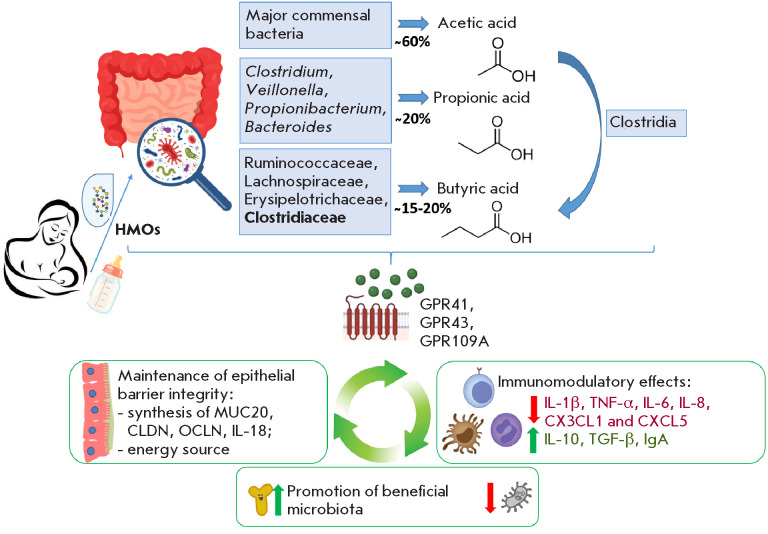
Protective mechanisms of SCFAs in the neonatal gut

Thus, SCFAs, particularly butyric acid, act not only as universal energy substrates for intestinal cells but also as potent regulators of local immunity, barrier function, and microbial homeostasis (Fig. 2). The impact of SCFAs is of particular significance for very preterm infants. This is due to an elevated risk of colonization by pathogenic microorganisms stemming from the underdeveloped intestinal barrier and immune system. This results in metabolites being key factors in the prevention and treatment of conditions such as NEC.

## SCFAs IN THE PATHOGENESIS OF NEC

Gut dysbiosis is recognized as one of the key factors in the pathogenesis of NEC [90]. Numerous studies conducted on model organisms have confirmed the significance of the microbiota and its metabolites in the development of this disease [91, 92]. In particular, the colonization of germ-free mice with bacteria isolated from the feces of NEC patients provokes NEClike intestinal injury [15].

Gut dysbiosis leads to impaired SCFA synthesis, which is particularly relevant for very preterm infants [93], whose minimal SCFA levels at birth gradually increase with post-conceptional age in the absence of NEC [94, 95, 96, 97]. However, SCFA metabolism in very preterm infants remains poorly understood.

**Table 3:** Characteristics of the gut microbiome and SCFAs in NEC in preterm infants

References	Clinical groups	Gestational age, weeks	Day of NEC onset	Predominant microorganisms, phylum and share (%, control vs NEC)	SCFAs, direction of change and fold change
Liu X.C. et al., 2022 [30]	NEC 7.0 ± 7.6 days prior (n = 17) and at manifestation (n = 12)	30.5 ± 2.1	30.2 ± 15.9	Proteobacteria ↑ (from 40 to 53%) Firmicutes ↓ (from 55 to 35%) Actinobacteriota ↑ (from 5 to 10%) Bacteroidota ↑ (from 0.5 to 4%)	*Acetic 1.8↓ **Propionic 1.2↓ **Butyric 1.1↓ **Isovaleric 2.3↓ *Total SCFAs 2.4↓
	Control (n = 17)	30.5 ± 1.9	–		
He Y. et al., 2021 [15]	NEC (n = 81)	31.0 (29.4–33.7)	15 (12–19)	***Proteobacteria ↑ (from 27 to 55%) ***Firmicutes ↓ (from 57 to 37%) ***Actinobacteriota ↓ (from 4 to 1%) ***Bacteroidota ↓ (from 10 to 3%)	**Butyric 1.4↓
	Control (n = 81)	31.1 (29.3–33.2)	–		
Xiong J., 2022 [98]	NEC (n = 22)	35.5 ± 2.2	11.6 (6.8–16.0)	Proteobacteria ↓ (from 50 to 37%) Firmicutes ↑ (from 45 to 57%) *Actinobacteriota↑ (from 3 to 5%) **Bacteroidota ↓ (from 4 to 1%)	**Acetic 1.5↓ **Propionic 2.3↓ **Butyric 2.7↓ **Isovaleric 2.0↓ **Caproic 2.7↑ **Total SCFAs 1.6↓
	FPIAP (n = 21)	36.5 ± 1.4	15.2 (11.0–22.0)		
Casaburi G. et al., 2023 [100]	NEC (n = 3)	~29	–	Proteobacteria ↑	*Formic 6.7↑
	Control (n = 10)	~29	–		
	NEC, treatment (n = 3)	~29	after 3 weeks of treatment	Proteobacteria ↓ Bacteroidetes ↑ compared to NEC onset	–
Huang H., 2022 [99]	NEC (n = 9)	31.6 (28.35–37.45)		Proteobacteria ↑ (from 30 to 65%) Firmicutes ↓ (from 65 to 30%) Actinobacteriota ↓ (from 5 to 1%) Bacteroidota ↑ (from 0 to 5%)	Not performed
	Control (n = 10)	37.75 (32.03–39.05)			
Pourcyrous M. et al., 2014 [101]	Formula (n = 9)	27	–	Not performed	**Total SCFAs 1.9↑ **Acetate 3.1↑ **Propionate 3.4↑
	Expressed milk (n = 10)	27	–		

Note: Bold text indicates changes of more than 2-fold in fecal SCFA levels in the NEC group compared to the control group.

^*^– p < 0.05,

^**^– p < 0.01,

^***^– p < 0.001 – for paired comparison of the NEC group and the control or comparison group.

Clinical research on the gut microbiota and generated SCFAs in very premature infants with NEC is still limited. Data from 16S rRNA sequencing in studies from 2021 to 2023 (Table 3) [15, 30, 98, 99, 100] validated the finding that Proteobacteria is the dominant bacteria in the immature gut microbiota, which has been linked to the development of NEC [27, 28, 29, 31]. Concurrently, a marked reduction in Firmicutes representatives, including major butyrate-producing strains, was observed prior to the onset of disease clinical manifestations [30].

A significant reduction in SCFA levels (p < 0.05) was found in newborns with NEC, especially formic, propionic, butyric, isovaleric, and caproic acids [15, 30, 98, 100]. Notably, in extremely preterm infants receiving expressed breast milk, fecal SCFA concentrations were significantly higher than in formula-fed infants [101]. While further investigation is needed to establish a direct causal relationship between SCFA levels and the onset of NEC, the results suggest that these metabolites may offer protection by preserving gut barrier integrity and regulating the inflammatory response.

However, significant variability in absolute SCFA concentrations has been revealed. For example, Liu X.C. et al. (2022) identified the mean fecal butyrate level in the control group as 41 µg/g [30], while in the study by He Y. et al. (2021), this figure was found to reach 225 µg/g [15]. These inconsistencies may be attributed to variances in the analytical techniques employed, the characteristics of the cohort, and the procedures for biomaterial collection and storage.

According to the reviews by Alsharairi N.A. et al. (2023) and Cifuentes M.P. et al. (2024), the function of butyrate in the development of neonatal NEC is still being debated [93, 102]. However, a detailed analysis of clinical works (Table 3) demonstrates a pronounced decrease in fecal butyrate levels in extremely preterm infants one week prior to the appearance of clinical NEC symptoms and at the onset of the disease, confirming its potential diagnostic value [15, 30]. For adults, butyric acid has also been linked to a lower chance of developing inflammatory bowel diseases, including ulcerative colitis and Crohn’s disease [103, 104]. Studies focusing on experiments highlight that butyrate improves the function of the intestinal barrier and lowers the inflammatory reactions of immune cells.

At the same time, a number of model experiments have revealed a negative role for butyrate in NEC [92, 105, 106, 107, 108]. Incomplete carbohydrate digestion in the small intestine leads to their fermentation in the colon with the formation of SCFAs, lactate, and gases such as carbon dioxide, methane, and hydrogen [109]. In preterm piglets modeling NEC, excessive formation of bacterial metabolites due to high levels of undigested lactose can trigger an inflammatory reaction [108].

The diverse effects of butyrate may be accounted for by its dose-related activity, as previously noted by Lin et al. (2002) [106]. Later, high concentrations of butyrate (greater than 16 mM) were confirmed to stimulate the synthesis of the pro-inflammatory cytokine IL-6, while low doses exert a protective effect by reducing IL-6 and NF-kB expression and enhancing the synthesis of the tight junction protein claudin-7 [57, 110]. Using a neonatal mouse NEC model, it was shown that an optimal level of butyric acid exists at which the risk of developing NEC is minimized [111].

The inconsistencies in data comparison across studies stem from the diversity of analytical methodologies, limited sample sizes of very premature infants, and the heterogeneity of NEC experimental models. Multicenter randomized trials using a unified methodology for biomaterial collection and analysis of microbiological and metabolic data may help clarify the role of SCFAs in NEC pathogenesis, as well as to determine optimal therapeutic doses for these metabolites. At the same time, GC-MS application for quantifying SCFAs in feces provides an accurate, noninvasive, rapid, and cost-effective method that can be readily implemented in routine neonatal practice for early diagnosis and monitoring of metabolic activity within the intestinal microflora of very preterm infants [102].

## CONCLUSION

A crucial step in the successful adaptation to life of a newborn child is the development of a symbiotic gut ecosystem. The formation of a dysbiotic microbial signature is influenced by several factors: birth at a low gestational age, congenital immaturity of the gut, frequent cesarean sections, extended stays in intensive care, antibiotic treatment, absence of contact with maternal microflora, and the lack or insufficiency of breast milk. The gut microbiome of very preterm infants is characterized by a predominance of Gram-negative bacteria of the phylum Proteobacteria, reduced microbial diversity, and overall instability. Research has correlated these alterations with an increased risk of life-threatening conditions, such as NEC.

Recent findings emphasize the critical role of microbiota metabolites, such as SCFAs, in maintaining the metabolic and immune homeostasis of the gut. However, the majority of the data has been obtained using murine models and cell lines. Moreover, accumulating evidence indicates that the SCFA metabolism operates within a dynamic equilibrium, and imbalances, whether elevated or diminished, can detrimentally affect health.

A more thorough understanding of the function of SCFAs and the microbiome in the development of NEC necessitates multicenter research encompassing a suitable number of cases of this rare disease. The incorporation of multi-omics techniques, encompassing metagenomic sequencing, transcriptomics, and targeted metabolomics, is necessary to identify particular microbial communities and metabolic biomarkers, including SCFAs. The improvement of rapid and non-invasive methods for functional microbiome profiling for clinical settings will lead to the creation of a system for assessing the risk of NEC development in very preterm infants. The correlation between neonatal pathologies and microbial metabolites presents opportunities for developing intricate pro- and postbiotic formulations.

## Abbreviations

GC: gas-chromatography
LC: liquid-chromatography
SCFA: short-chain fatty acids
MS: mass-spectrometry
NEC: necrotizing enterocolitis
NICU: neonatal intensive care unit
PCR: polymerase chain reaction
